# Criteria for effective design, construction, and gene knockdown by shRNA vectors

**DOI:** 10.1186/1472-6750-6-7

**Published:** 2006-01-24

**Authors:** Debra J Taxman, Laura R Livingstone, Jinghua Zhang, Brian J Conti, Heather A Iocca, Kristi L Williams, John D Lich, Jenny P-Y Ting, William Reed

**Affiliations:** 1 Department of Microbiology and Immunology, Lineberger Comprehensive Cancer Center; University of North Carolina, Chapel Hill, NC 27599, USA; 2 Program of Molecular Biology and Biotechnology; University of North Carolina, Chapel Hill, NC 27599, USA; 3 Department of Biochemistry and Biophysics, University of North Carolina, Chapel Hill, NC 27599, USA; 4 Center for Environmental Medicine, Asthma and Lung Biology, University of North Carolina, Chapel Hill, NC 27599, USA

## Abstract

**Background:**

RNA interference (RNAi) technology is a powerful methodology recently developed for the specific knockdown of targeted genes. RNAi is most commonly achieved either transiently by transfection of small interfering (si) RNA oligonucleotides, or stably using short hairpin (sh) RNA expressed from a DNA vector or virus. Much controversy has surrounded the development of rules for the design of effective siRNA oligonucleotides; and whether these rules apply to shRNA is not well characterized.

**Results:**

To determine whether published algorithms for siRNA oligonucleotide design apply to shRNA, we constructed 27 shRNAs from 11 human genes expressed stably using retroviral vectors. We demonstrate an efficient method for preparing wild-type and mutant control shRNA vectors simultaneously using oligonucleotide hybrids. We show that sequencing through shRNA vectors can be problematic due to the intrinsic secondary structure of the hairpin, and we determine a strategy for effective sequencing by using a combination of modified BigDye chemistries and DNA relaxing agents. The efficacy of knockdown for the 27 shRNA vectors was evaluated against six published algorithms for siRNA oligonucleotide design. Our results show that none of the scoring algorithms can explain a significant percentage of variance in shRNA knockdown efficacy as assessed by linear regression analysis or ROC curve analysis. Application of a modification based on the stability of the 6 central bases of each shRNA provides fair-to-good predictions of knockdown efficacy for three of the algorithms. Analysis of an independent set of data from 38 shRNAs pooled from previous publications confirms these findings.

**Conclusion:**

The use of mixed oligonucleotide pairs provides a time and cost efficient method of producing wild type and mutant control shRNA vectors. The addition to sequencing reactions of a combination of mixed dITP/dGTP chemistries and DNA relaxing agents enables read through the intrinsic secondary structure of problematic shRNA vectors. Six published algorithms for siRNA oligonucleotide design that were tested in this study show little or no efficacy at predicting shRNA knockdown outcome. However, application of a modification based on the central shRNA stability should provide a useful improvement to the design of effective shRNA vectors.

## Background

RNA interference (RNAi) is a naturally occurring phenomenon by which RNA duplexes known as short interfering RNA (siRNA) can reduce gene expression through enzymatic cleavage of a target mRNA mediated by the RNA-induced silencing complex (RISC). The ability of synthetic siRNA to inhibit targeted genes with near specificity makes it an extremely powerful tool for functional genomics that has drawn considerable interest recently [1,2]. RNAi is commonly achieved by introducing chemically synthesized siRNA 19–22 mers into cells by transfection. However, many cells and cell lines are either refractory to or adversely affected by transfection, and the transient nature of this methodology renders it unsuitable for the generation of long-term cell lines of the desirable phenotype. Two alternatives to synthetic siRNA are DNA-vector mediated RNAi production [3-5], and most recently viral-mediated siRNA synthesis [6-10]. For the latter technologies, sense and antisense strands can be expressed from different promoters [11]. Alternatively, short hairpin (sh) RNAs, expressed from a single promoter, are processed into siRNAs by Dicer or a homologous double strand RNase [12].

One caveat of siRNA design is that not all 19–22 base RNA duplexes will cleave their target with efficacy, and much effort has gone towards identifying a set of rules for selecting an effective siRNA target site within a gene. Recent findings [13,14] offered the first clue towards the development of guidelines for selecting an siRNA target site. These studies showed that the RISC complex is asymmetric and favors the strand of the siRNA duplex with the least thermodynamically stable 5' terminus. Subsequently, Reynolds et al. designed an algorithm based on statistical data showing patterns of efficacy for siRNA oligonucleotides containing specific residues at defined positions within the 19-mer [15]. A limitation of their study is that a small number of genes were tested. Several additional algorithms for designing effective siRNAs have been published since those initial reports with surprisingly disparate results, making the determination of which residues are generally favorable for siRNA efficacy a point of controversy [16-20]. Additionally, whether any of the algorithms developed for synthetic siRNA oligonucleotides apply to the design of shRNA expressed stably from a vector has not been well explored.

In the present report, we construct and analyze a set of 27 shRNAs for 11 different human genes. To our knowledge this is the largest individual set of data published for shRNA 19-mers. We describe a method for simultaneously preparing wild type and control mutant shRNA vectors that is time and cost efficient, and show that sequencing of shRNA plasmids can be quite problematic due to the intrinsic secondary structure of the hairpin. We examine several different strategies for overcoming this problem including the use of modified BigDye chemistries and the addition of agents known to relax DNA structure. The knockdown efficacy for each of the 27 shRNAs was evaluated against six published algorithms for siRNA oligonucleotide design by linear regression and ROC curve analyses. We describe a modification of three of the algorithms that provides fair-to-good prediction of shRNA efficacy, and confirm the significance of the modified algorithms using a pooled set of shRNAs from previous publications. These findings should be of general applicability in the design and construction of shRNA vectors.

## Results and discussion

### Design and preparation of shRNA plasmids

To address the question of how shRNA sequence correlates with knockdown efficacy, 27 shRNA vectors from 11 different genes were designed and constructed (Table 1). Target sequences were selected in the coding region of each gene and were designed to broadly conform to the seminal studies of sequence features for siRNA oligomer efficacy [13-15]. Accordingly, sequences are low in runs and have a G/C ratio of about 50%. The shRNAs were designed to target sites that are devoid of single nucleotide polymorphisms, and correspond to all splice variants amplified by our real time PCR primer sets.

**Figure 1:**
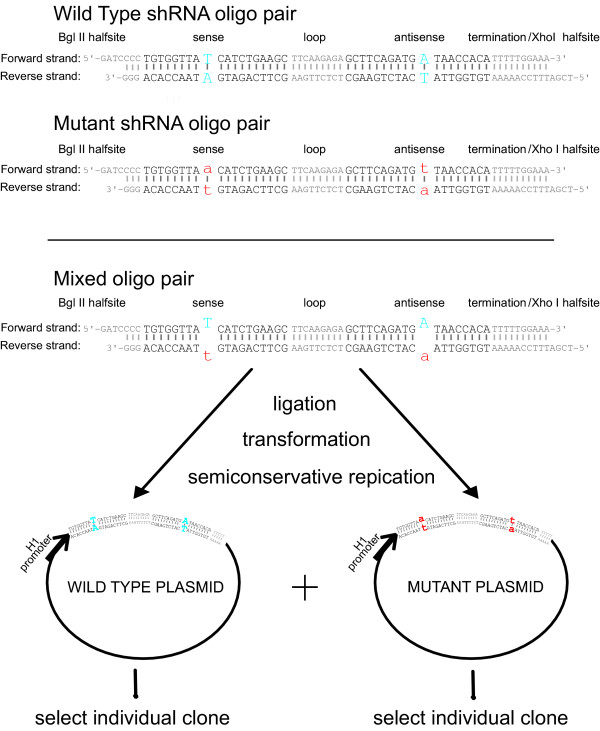
**Design for producing wild-type and mutant shRNA vectors simultaneously**. A forward strand of the wild-type hairpin (blue) is synthesized together with a reverse strand containing a one bp mutation within both the sense and antisense copy of the target sequence (shown in red). The double stranded hybrid is ligated into the retroviral vector 5' of an H1 promoter and transformed into competent bacteria. Since replication is semi-conservative, the daughter bacteria will be of two different populations that carry either a double-stranded wild-type or a double-stranded mutant vector and can be isolated by preparing and sequencing individual colonies.

**Figure 2:**
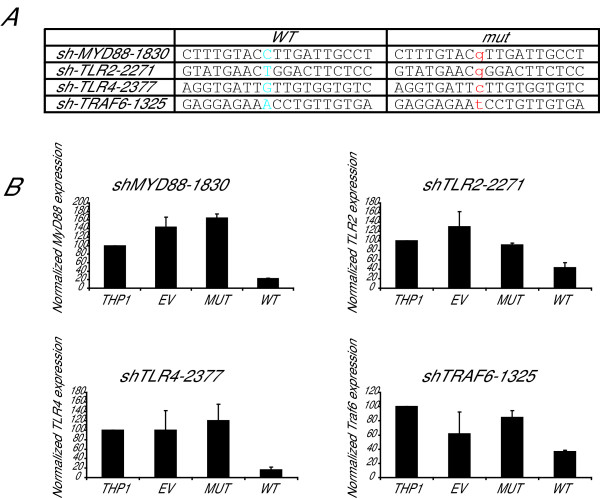
**Gene expression analysis for wild-type and mutant shRNA vectors prepared simultaneously using wild-type/mutant double stranded hybrids**. (A) Sequences of the target sites for four wild-type and mutant shRNA vectors that were prepared simultaneously as detailed in Figure 1. (B) Realtime analysis of shRNA knockdown, and loss of knockdown by mutant shRNA vectors from (A). Values are standardized to 100% in non-transduced THP1 cells. The expression in THP1 cells transduced with an empty vector (EV) is shown as an additional control. Values represent average +SEM for at least three assays performed in duplicate.

Since siRNAs can have off-target effects, it is important for functional assays to make a specific mutant with one or more base mismatch within the target recognition site as a control [21]. To conserve time and cost, we have developed a method of making wild-type and mutant shRNA vectors simultaneously (detailed in Methods and Figure 1). Gene knockdown results for four wild-type/mutant shRNA pairs are shown in Figure 2. These results demonstrate the utility of this method in providing a point mutant shRNA vector that can serve as a loss-of-function control for gene knockdown by wild type shRNAs. Though detailed protocols have been published for construction of shRNA vectors [22], this is the first protocol for producing wild-type and mutant vectors simultaneously and should facilitate the implementation of highly controlled system for shRNA.

### Strategy for accurate sequencing through hairpin structures

Verifying the sequence of an shRNA hairpin is essential since mismatch of even one nucleotide within the target sequence can ablate knockdown (Figure 2 and [5,23].) An issue that is frequently encountered in the preparation of shRNA vectors is that many are difficult to sequence due to the intrinsic secondary structure of the hairpin. One strategy recently proposed to overcome this issue involves engineering a restriction site within the loop/stem region of the hairpin to physically separate the inverted repeats by digestion, and then piecing together sequence using sense and antisense primers [24]. However, the ability to achieve sequencing of shRNA constructs without modifying stem/loop sequence would be of clear advantage. To address this possibility, we evaluated modified sequencing reactions for improvement in the read-through of the hairpin secondary structure in three shRNA hairpins. Modifications include adding agents known to relax DNA structure including DMSO, Betaine, PCRx Enhancer and ThermoFidelase I; and adding increasing amounts of dGTP BigDye terminator (dGTP) chemistry to the standard BigDye v1.1 (BD) chemistry which contains dITP rather than dGTP.

**Figure 3:**
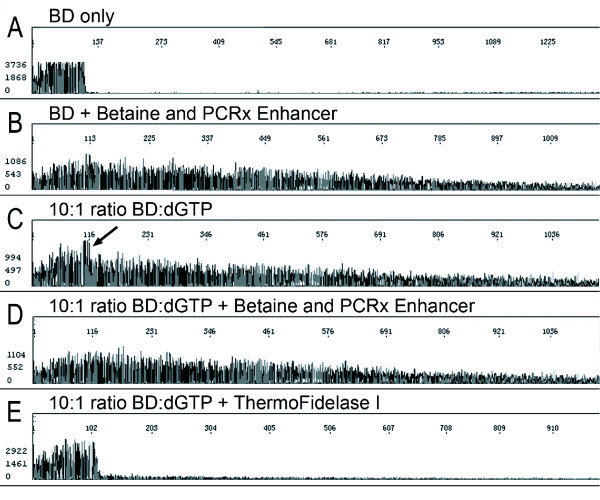
**DNA sequencing of pHSPG-shTLR4 using modified reaction conditions**. DNA sequencing peaks are shown in a full scale view where base positions are indicated by the row of numbers in each panel and the Y axis is the signal intensity. Sequencing reaction conditions shown are BigDye v1.1 (BD) chemistry (A), 0.83 M Betaine + 1 ×PCRx Enhancer in BD chemistry (B), 10:1 BD:dGTP chemistries (C), 0.83 M Betaine + 1 ×PCRx Enhancer in 10:1 BD:dGTP chemistries (D), and 1 × ThermoFidelase I in 10:1 BD:dGTP chemistries (E). The drop in signal (step in peak height) at the hairpin is highlighted by an arrow in the 10:1 BD:dGTP chemistries panel.

Sequencing results for each of the three DNA constructs are summarized in Table 2. Read-through of the hairpin structure was measured as the ratio of the peak height about 300 bases after the hairpin structure to the signal about 50 bases before the hairpin structure. A ratio of 1 indicates no loss in signal and 0 indicates complete loss of read-though. In the absence of any additive to BD chemistry, the hairpin caused a reduction in peak height ratio for our less tightly structured hairpin, pHSPG-shmutTLR4, to 0.4, and a complete loss in read through for the other two plasmids. This can be visualized as an abrupt stop in the sequence peak profile for pHSPG-shTLR4 (Figure 3A).

Among the DNA relaxing agents, 5% DMSO, 0.83 M Betaine and 1 × PCRx Enhancer each improved the sequence read significantly for some constructs. However, the addition of 0.83 M Betaine plus 1 × PCRx Enhancer to BD chemistry was found to sequence most consistently, with peak height ratios of 0.5–0.9 (Table 2 and Figure 3B). The addition of 10:1 BD:dGTP chemistries alone also improved read through somewhat, with peak height ratios of 0.5–0.6 (Table 2 and Figure 3C). The sub-optimal peak height ratio for 10:1 BD:dGTP can be attributed to a visible step in the sequence peak profile after the secondary structure region where the signal is reduced (Figure 3C, arrow). Increasing the dGTP chemistry content to 5:1 and 3:1 BD:dGTP or using straight dGTP chemistry increased the peak height ratio and reduced the step somewhat (0.6 to 0.8 ratio). However, the mixed incorporation of dITP and dGTP resulted in worse peak broadening as the amount of dGTP used increased [see Additional file 1], and dGTP only chemistry caused severe sequence compressions (data not shown). The best overall results were observed by combining Betaine plus PCRx and 10:1 BD:dGTP mixed chemistries together. This combination reduced the step with less peak broadening and increased peak height ratios to 0.9–1.0 (Table 2 and Figure 3D). ThermoFidelase I, a DNA destabililizing enzyme that is frequently used to improve sequencing of genomic DNA [25,26], did not improve sequencing of any of the three hairpins in straight BD chemistry (data not shown), and actually reduced the peak height ratio significantly in 10:1 BD:dGTP chemistries for all three shRNA constructs, causing the reappearance of a stop at the hairpin structure (Table 2 and Figure 3E).

In summary, the combination of 10:1 BD:GTP chemistries, 0.83 M Betaine, and 1 × PCRx Enhancer provided optimal sequencing, and mixed BD:dGTP chemistries, Betaine, PCRx Enhancer, and DMSO each had some positive effects on their own. ThermoFidelase I, however, probably should be avoided for shRNA vectors with difficult intrinsic secondary structure.

### Correlation between shRNA knockdown efficiency and published algorithms for siRNA design

To determine whether the efficacy of knockdown by shRNA vectors correlates with published rules for the design of effective siRNA oligonucleotides, shRNAs were evaluated for their ability to knockdown gene expression. The shRNAs were transduced stably into either THP1 or Jurkat human cell lines as detailed in Table 3, first two Columns. The average knockdown was determined from RNA collected on three or more different days and is listed for each shRNA (Column 3). Knockdown was shown to be reproducible for cell lines that were independently transduced and sorted, suggesting that knockdown is a function of the shRNA target sequence rather than features of the viral transduction [see Additional file 2]. More than one third of the shRNA vectors constructed were unable to suppress transcription (<10% in Column 3), despite comparable growth rates and long term expression of the GFP marker at high levels in these cell lines. Furthermore, great variations in knockdown efficacy for several shRNAs made against many of the same genes (i.e., *CLR16.2*, *CLR19.3 *and *TLR4*) argue against any simple biological reasons for differences in efficacy for these genes. Many of the ineffective shRNAs have negative 5' ΔΔG values and high Reynolds scoring, each which have been hypothesized to correlate with siRNA knockdown efficacy (Table 3, Columns 4 and 5) [13-15]. Conversely, among the shRNAs that were able to confer gene knockdown, several had either positive 5'ΔΔG values or low Reynolds scores. These findings indicate that 5'ΔΔG and Reynolds scoring algorithm for siRNA may not provide positive correlative criteria for shRNA design.

**Figure 4:**
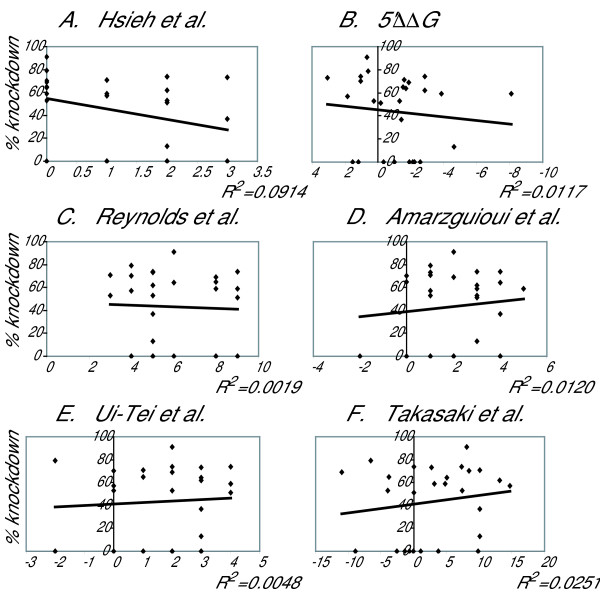
**Correlation between shRNA knockdown efficacy and scoring for six published algorithms for siRNA**. Algorithm scores for each shRNA target site from Table 2 are plotted against observed knockdown efficiency for the Hsieh et al. (A), 5' ΔΔG (free energy differential) (B), Reynolds et al. (C), Amarzguioui et al. (D), Ui-Tei et al. (E) and Takasaki et al. (F) algorithms. The 5' ΔΔG score is plotted on a reverse horizontal axis since knockdown efficacy is predicted to correlate with negative 5' ΔΔG value. A trend line is shown along with the R^2 ^value for each plot. Knockdown of less than 10% is plotted as zero.

To determine whether other published algorithms for siRNA oligonucleotide design can be applied to shRNA vectors, each of the shRNA target sites was evaluated by four additional algorithms, and scores were plotted against the percent knockdown for each shRNA (Table 3, Columns 6–9 and Fig. 4). For each algorithm plot a best fit line was drawn and the R^2 ^value calculated as an indication of whether the variance in knockdown efficacy can be explained by the algorithm scoring. Results confirm a poor association between shRNA efficacy and either 5' ΔΔG (free energy differential) considerations [13] or the Reynolds et al. algorithm [15], and also demonstrate a poor association with the Hsieh et al. algorithm [19], with each in fact showing a weak reverse correlation with the data. The algorithms of Amarguizoui et al. [20], Ui-Tei et al. [18], and Takasaki et al. [17], correlate directly with shRNA efficacy. However, none of the algorithm scores explain a significant percentage of the variance in knockdown efficacy. Among the algorithms tested, the Takasaki et al. scoring system shows the highest association, with an R^2 ^value of 0.0251.

Because these results suggest that a linear relationship does not strongly apply to shRNA knockdown for any of the six algorithms, we evaluated each of the algorithms by ROC curve analysis to determine whether any algorithm is superior to the others at identifying effective shRNAs. The ROC curve is a plot of sensitivity (the true positive fraction, TPF) versus 1 minus the specificity (the false positive fraction, FPF) that is generated by varying the decision threshold between the minimum and maximum algorithm score. The diagonal of the ROC plot represents the ROC curve for an algorithm that is no better at discrimination than random selection. Algorithms that are poor discriminators have ROC curves that track along the diagonal and have an area under the ROC curve (AUC) that is not significantly different from the AUC of the diagonal (0.5). Algorithms that are good discriminators have ROC curves with strong convex deviation from the diagonal and AUCs that approach 1 and are significantly different from the AUC of the diagonal.

**Figure 5:**
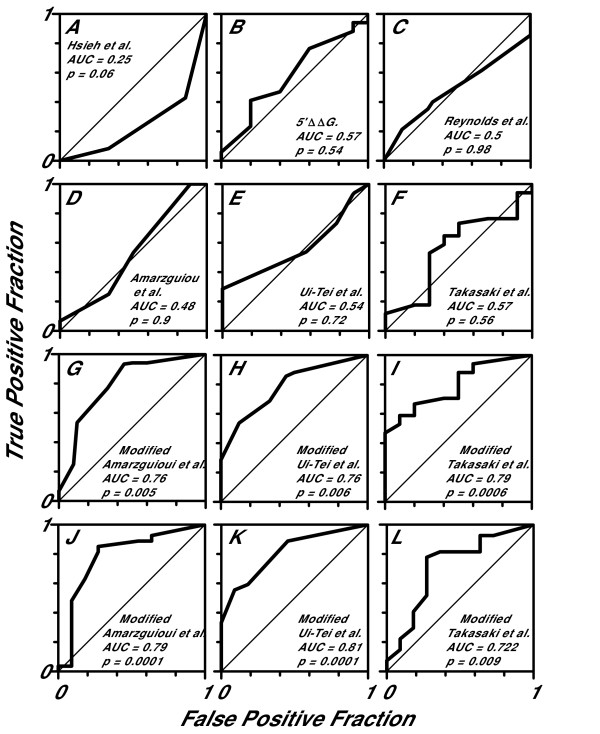
**ROC curve analysis of siRNA scoring algorithms**. The true positive fraction was plotted against the false positive fraction as the decision threshold varied from minimum to maximum scores (see Materials and Methods for details) for the Hsieh et al. (A), 5' ΔΔG (free energy differential) (B), Reynolds et al. (C), Amarzguioui et al. (D), Ui-Tei et al. (E) and Takasaki et al. (F) algorithms using an efficacy threshold of 50% knockdown. ROC curves for modified Amarzguioui et al. (G), Ui-Tei et al. (H) and Takasaki et al. (I) algorithms are also shown. A set of 38 published shRNAs (Table 5) was analyzed using the modified Amarzguioui et al. (J), Ui-Tei et al. (K) and Takasaki et al. (L) algorithms to confirm the utility of the modified algorithms. The area under the curve (AUC) and the probability (p) that the AUC is significantly different from 0.5, the area under diagonal, is indicated for each ROC curve.

The Hsieh et al. algorithm had a concave ROC curve (Fig. 5A) indicating unacceptable sensitivity and specificy in discriminating effective from ineffective shRNAs. The ROC curves for all other algorithms (Figs. 5B–F) tracked near the diagonal of the ROC plot and had AUCs that were not significantly different from the AUC of the diagonal (Figs 5B–F). Thus, none of the algorithms showed a statistically significant ability to discriminate between effective and ineffective shRNAs.

The Takasaki et al. algorithm (Fig. 5F) showed the most promise as a discriminator of effective from ineffective shRNAs. However, this algorithm suffered from a relatively high false positive fraction for decision thresholds near the maximum score as indicated by the weak, erratic deviation from the diagonal near the origin of the ROC curve (Fig. 5F). This indicated that the algorithm assigned a high score to a number of ineffective shRNAs. Inspection of the data revealed that two of the three high-scoring ineffective shRNAs targeted genes whose expression was successfully knocked-down by other shRNAs (Table 3, asterisks). Thus it is unlikely that the inefficacy of the shRNAs is a consequence of selective pressure against the stable suppression of gene expression. It is more likely that the Takasaki et al. algorithm does not account for a critical feature of effective shRNAs.

### Application of an algorithm modification based on the stability of the 6 central bases of each shRNA

Inspection of the physical properties of the high scoring ineffective shRNAs revealed that the average stability of the duplex formed by the 6 central bases of the shRNAs (bases 6–11 of the sense strand hybridized to bases 9–14 of the antisense strand) was greater than the average stability of high scoring effective shRNAs (ΔG = -13.1 ± 0.1 versus -11.1 ± 1 kcal/mol respectively). Based on this observation, the Takasaki et al. algorithm was modified such that shRNAs with a central duplex ΔG equal to or less than -12.9 kcal/mol were assigned a minimum score (Table 4). This modification assigned minimum scores to five shRNAs, four which were ineffective, thus increasing the specificity of the algorithm without a significant loss in sensitivity. A minimum score assigned to one effective shRNA (71% knockdown), indicates that other properties in addition to central duplex stability influence efficacy. Nevertheless, the addition of this modification eliminated the weak erratic deviation of the ROC curve from the diagonal for high decision thresholds and increased the AUC to 0.79 (Fig. 5I). Similar modification of the Amarzguioui et al. and Ui-Tei et al. algorithms also raised the AUCs of their ROC curves (Figs. 5G and 5H). With this modification, the AUCs of the ROC curves for all three modified algorithms were significantly different from the AUC of the diagonal (Figs. 5G–I), indicating statistically significant predictive capability. Differences between AUCs of the ROC curves for the modified algorithms were not significant, so on statistical grounds all three of the modified algorithms were of equal utility. The 5' ΔΔG, Reynolds et al, and the Hsieh et al. algorithms were not improved to a statistically significant predictive capability by applying the central duplex ΔG modification (data not shown).

To address the possibility that the improvement achieved by the modification of the Amarzguioui et al, Ui-Tei et al, and Takasaki et al. algorithms is a consequence of overfitting our set of shRNAs, an independent set of 38 shRNAs pooled from previous publications ([18,27-33]; Table 5) were subjected to analysis. While none of the ROC curves for the three unmodified algorithms had an AUC significantly different from that of the diagonal (Amarzguioui et al., p = 0.174; Ui-Tei et al. p = 0.09; Takasaki et al., p = 0.26), all of the modified algorithms yielded ROC curves with AUCs significantly different from the AUC of the diagonal (p = 0.0001–0.009; Figs. 5J–L). On statistical grounds, all three of the modified algorithms were of equal utility as the AUCs of the ROC curves for the modified algorithms were all significantly different from the AUC of the diagonal, but not significantly different from each other. This analysis of an independent set of shRNAs suggests that the modification of the algorithms is of general validity.

Because minimizing the false positive rate is the primary concern in shRNA design, we recommend using the modified Ui-Tei et al. algorithm, which had the lowest high false positive fraction at decision thresholds near the maximum score as indicated by the strong deviation from the diagonal near the origin of the ROC curve (Figs. 5H and 5K). Using a decision threshold of 3 limits selection of shRNAs to a region of the ROC curve where the sensitivity was acceptable (0.28–.33), while the specificity was very good (1.0). By setting this decision threshold, the false positive fraction was minimized, while 28 – 33% of the effective shRNAs were identified from our shRNAs and the published set of shRNAs respectively. Should the sensitivity need to be increased, we recommend using a decision threshold of 2. This threshold had a sensitivity of 0.54 – 0.55 and a specificity of 0.88 – 0.9. If the decision threshold was further relaxed to 0, the sensitivity increased to 0.86 – 0.9, but the specificity fell to 0.55 – 0.54. We recommend using the highest of these decision thresholds possible.

Though statistically small, this study has the advantage to our knowledge of being the largest published set of 19-mer based shRNAs to date. In addition, unlike other shRNA studies that are necessarily skewed toward effective shRNAs, our study includes both functional and non-functional shRNAs. We have shown that modified Ui-Tei et al., Amarzguioui et al. and Takasaki et al. algorithms are fair to good predictive tools that distinguish effective from ineffective shRNAs. However, significant shortcomings still exist in the modified algorithms. A direct assessment of the algorithm modifications using shRNAs designed according to each original and modified algorithm would lend support to these findings. These algorithms are meant to reduce the number of false positive shRNAs selected, not completely eliminate them altogether, and thus this would require a large number of shRNAs to obtain a statistically significant difference in false positive rate. The availability of larger shRNA data sets should support the development of algorithms with improved sensitivity and specificity. Additionally, several software applications for siRNA oligonucleotide design that were not considered in this study may be of use in the design of shRNAs [16,34-36]. Criteria for designing functional siRNA oligonucleotides remain controversial as evidenced by the large number of studies still being devised for siRNA design, and since we did not test these sequences as siRNAs it cannot be established whether the modification of these algorithms also applies in the context of siRNA oligonucleotides. shRNA has an added layer of complexity over siRNA oligonucleotides since the hairpin needs to be processed within the cell before entering the RISC complex. Moreover, selective pressure against the stable expression of shRNAs that are deleterious to cell growth would be expected to lend an additional constraint to the stable expression of certain shRNAs. Despite these complexities, our findings begin to bring insight into the ability to apply siRNA algorithms for design of functional shRNAs.

## Conclusion

We have provided several important strategies that should facilitate the generation of effective shRNA vectors for gene knockdown in mammalian cells. The ability to produce wild-type and mutant shRNA vectors simultaneously using mixed oligonucleotide pairs provides an efficient method to generate a specific control vector with little added time or cost. This strategy should be particularly useful in generating specific controls in high throughput applications. Difficulty in sequencing through the high intrinsic secondary structure of some hairpin vectors also has presented a major constraint in the construction of shRNA vectors, and the knowledge that sequencing issues can be resolved by modifying BigDye chemistries and adding Betaine and other DNA relaxing agents should be valuable regardless of the method of shRNA design and construction. Using data from 27 shRNAs that we have constructed we have performed an analysis of the ability of published algorithms for siRNA oligonucleotide target selection to predict knockdown efficacy. Our results show that shRNA efficacy cannot strictly be explained by any of the six algorithms tested. We provide a modification, however, that greatly improves the predictability of the Ui-Tei et al., Amarzguioui et al. and Takasaki et al algorithms. Results were confirmed using data from 38 previously published shRNAs. These findings should be of significant applicability in the design and preparation of functional shRNAs.

## Methods

### Cell lines and cell culture

THP1 monocytic cell and Jurkat T cell lines were cultured in RPMI, 10% FCS. Cultures were maintained between 2 and 8 × 10^5 ^cells/ml and standardized to equivalent densities before assessing knockdown efficiencies.

### Plasmid design and construction

Retroviral vectors for shRNA expression have a pHSPG backbone [37] with an inserted H1 RNA promoter driving shRNA expression. The pHSPG vector also has a green fluorescent protein (GFP) gene driven by a phosphoglycerate kinase promoter as a marker. The H1 promoter and shRNA expression cassette were inserted into the pHSPG vector by one of two methods. In the first method, a double stranded oligomer is synthesized with Bgl II and Xho I half sites on the ends. This is prepared as either a matched pair or a wild-type/mutant hybrid (Fig. 1). To prepare wild-type and mutant shRNA vectors simultaneously, a forward strand oligomer is synthesized that contains the wild-type hairpin. In parallel, a mutant reverse strand with a one bp mismatch within the target sequence is also synthesized. Despite the mismatches between the forward wild-type and reverse mutant strands, annealing can still occur efficiently under optimized conditions. The ds oligonucleoltide is annealed by combining 1000 pmol of each oligomer strand in 50 μl of annealing buffer (100 mM potassium acetate, 30 mM HEPES-KOH, pH 7.4, 2 mM Mg-acetate). The mixture is boiled for five minutes and then cooled slowly to 4°C. The annealed double stranded oligomer is ligated into Bgl II and Xho I half sites 3' of the H1 promoter that is inserted into the 3' long terminal repeat (LTR) of pHSPG generating a self-inactivating LTR. The double stranded hybrid is ligated into the vector 5' of a pol III promoter and is transformed into competent bacteria. Since replication is semi-conservative, the daughter bacteria will be of two different populations that carry either a double-stranded wild-type or a double-stranded mutant vector. Bacteria carrying either wild-type or mutant vectors can then be isolated from individual colonies and sequenced. Oligos used for this method had the sequence: GATCCCC-N19-TTCAAGAGA-rN19-TTTTTGGAAA; and TCGATTTCCAAAAA-N19-TCTCTTGAA-rN19-GGG (where N19 is the sense of the target sequence and rN19 is the antisense). We have routinely used DH5α to prepare wild-type and mutant shRNA vectors with approximately equal yields of each type of vector; however, a repair-deficient *E. coli *mutant could theoretically improve the efficiency of simultaneous construction.

A second design involves PCR using a primer complementary to the 5' end of the H1 promoter together with an shRNA-specific long-primer whose 3' end is complementary to the 3' end of the H1 promoter. PCR is performed using Pfx polymerase with PCRx enhancer (this combination has proved essential for reducing the number of mutations introduced within the amplified region). Oligos used for this method were: GCGGCCGCGATATCGAACGCTGACGTCATCAACCC (universal oligo); and TGCTCTAGAAAAA-N19-TCTCTTGAA-rN19-GGGAAAGAGTGGTCTCATACAGAACTTATAAGATTCC, where N19 is the sense of the target sequence and rN19 is the antisense. Sequences complimentary to the H1 promoter are underlined. PCR fragments were digested with EcoRV and XbaI and ligated into the 3' LTR of pHSPG. All constructs were verified by sequencing.

### Sequencing of shRNA vectors

DNA sequencing was done at the UNC-CH Genome Analysis Facility. Sequencing reactions were 12.5 uL total volume containing 1 × BigDye Terminator v1.1 Cycle Sequencing Ready Reaction Mix (Applied Biosystems), 0.26 ug of DNA and 3.75 pmole of primer. LTRa primer (sequence CGCGAACAGAAGCGAGAA) that binds the HSPG vector approximately 120 bp downstream from the inserted hairpin was used in all sequencing reactions. The shRNA vectors used to assess sequencing efficacy were constructed as stem loop hairpins as described above and contain the following target sequences: pHSPG-shTLR4, AGGTGATTGTTGTGGTGTC; pHSPG-shmutTLR4, AGGTGATTCTTGTGGTGTC; pHSPG-shmCNN3, AGGAATGAGCGTGTATGGG; and pHSPG-shTLR2, GTATGAACTGGACTTCTCC. Modified sequencing reactions substituted part or all of the BigDye v1.1 chemistry with ABI Prism dGTP BigDye Terminator Ready Reaction Mix (Applied Biosystems). Ratios of 20:1, 10:1, 5:1 and 3:1 BD:dGTP chemistries and straight dGTP chemistry were used. Additives evaluated in sequencing reactions were: 0.83 M Betaine (Sigma part # B-0300), 5% DMSO (Sigma part # D-2650), 1 × PCRx Enhancer (in Invitrogen kit part # 11495-017), 1 × (1 uL Thermofidelase/20 uL sequencing reaction) ThermoFidelase I (Fidelity Systems) and 10 × primer concentration. The thermal cycler protocol used for cycle sequencing was: 95'C for 3 minutes (or 5 minutes when using ThermoFidelase I) followed by 25 cycles of 98'C for 40 seconds (1^st ^cycle) or 10 seconds (subsequent cycles), 50'C for 5 seconds and 60'C for 4 minutes. Sequencing reactions were purified using Centri-Sep 96 well spin plates (Princeton Separations), and the purified reaction products were run on a 3730 DNA Analyzer (Applied Biosystems) with a 50 cm array using the LongRead protocol. As a measure of read through efficacy peak height ratios were determined about 300 bases after and 50 bases before the hairpin.

### Virus preparation, transduction and cell sorting

To prepare virus, pHSPG-shRNA plasmids were co-transfected into 293T cells with gag/pol and VSVg vectors by the calcium phosphate method. Viral supernatants were collected 24 and 48 hours following transfection and used to transduce THP1 or Jurkat cells by spinoculation. THP1 cells were transduced with virus on two consecutive days to increase transduction levels. Following approximately one week of culture, stably transduced cells were isolated by sorting for GFP. FACS analysis studies suggest that GFP expression is 95% stable for at least two months following sorting (not shown).

### RNA expression analyses

Total RNA was isolated with an RNeasy isolation kit (Qiagen) using the recommended protocol. To increase specificity, cDNA was reverse transcribed using oligo dT primer and Superscript III RT (GibcoBRL). Real-time PCR experiments were performed using an AB Prism 7700 instrument (Applied Biosystems) with 57°C annealing temperature. For *18s, CLR19.6/NALP11, CLR19.3/NALP12, MYD88, TLR2, TLR4, and TRAF6*, real-time PCR was performed using Absolute QPCR Mix (ABgene) mix and either TET or FAM labeled probes. The following are the sequences of the oligonucleotides used, listed as [forward; reverse; probe]: *18s*-[CGGCTACCACATCCAAGG; GCTGCTGGCACCAGACTT; Tet-CAAATTACCCACTCCCGACCCG-Tamra]; *CLR19*.6/NALP11-[TCAATGATGCGTAAGGAAAGA; ACTTTCCCATTGCAGCATGA; Fam-CTTTGCATGCCTCCTGATTGCGGT-Tamra]; *CLR19.3/NALP12*-[AGAGGACCTGGTGAGGGATAC; CTTCCAGAAGGCATGTTGAC; Fam-CCCGTCCTCACTTGGGAACCA-Tamra]; *MYD88*-[CTCTGTAGGCCGACTGC; CTGCTGCTGCTTCAAGATA; Fam-TGGCAATCCTCCTCAATGCTGGGTC-Tamra]; *TLR2*-[GGTCATCATCAGCCTCTCCA; GAGCTGCCCTTGCAGATAC; Fam-CCTCCAATCAGGCTTCTCTGTCTTGTGACC-Tamra]; *TLR4*: [AGAGCCTAAGCCACCTCT; CTAGAGATGCTAGATTTGTCTCCA; Fam-AGCCACCAGCTTCTGTAAACTTGATAGTCCAGA-Tamra]; *TRAF6*: [CCATGCGGCCATAGGTT; TTTCCAGCAGTATTTCATTGTCA; Fam-TGGACATTTGTGACCTGCATCCCTTATTGAT-Tamra]. For *ASC/PYCARD, CLR16.2, MAL/TIRAP, TRAM/TICAM2, and TRIF/ICAM1*, realtime PCR was performed using ABsolute SYBR green mix (ABgene) and the following primers, listed as [forward; reverse]: *ASC/PYCARD1*-[AACCCAAGCAAGATGCGGAAG; TTAGGGCCTGGAGGAGCAAG]; *CLR16.2*-[TCAACACAGCCCTCACTGCTCTCTATCTC; AGCCACCCCAATGGCATTTCCTCTTAAGTC]; *MAL/TIRAP*-[GGACTCATCTCCTGCCTAAC; CATGGTGAGGCCTGCAATCT]; *TRAM/TICAM2*-[GGCACAGTGTGGATACAAGT; ACATCTCTTCCACGCTCTGA]; *TRIF/TICAM1*-[CAGGAGCCTGAGGAGATGAG; GGGTAGTTGGTGCTGGTTTC]. Primers were designed to span exon/intron junctions where possible. All RNA expression analyses were done at least in triplicate for RNA isolated on different days and knockdowns were verified with at least one control hairpin. Values represent average observed knockdown for RNA from different days of cell culture and were standardized to 18s rRNA expression.

### Implimentation of algorithms

The free energy (ΔG) of RNA duplex formation for the 5 bases at the 5' end of the sense and antisense strands was determined using the thermodynamic parameters and expanded nearest-neighbor model of Xia et al. [38]. The 5' ΔΔG (differential free energy) was calculated by subtracting the ΔG of the antisense strand from that of the sense strand. Determination of scores for the Reynolds et al., Amarzgiuoui et al., and Takasaki et al. algorithms was as described [15,17,20]. The Hsieh et al. score represents the interpretation of the Hsieh et al. design criteria as published by Saetrom and Snove [16,19]. For the Ui-Tei algorithm sequences with a C or G on the 5' end scored 1 point, whereas those with an A or T scored -1 point. Sequences with an A or T on the 3' end scored 1 point, whereas those with a C or G scored -1 point. Sequences with 5 or more A or T bases in the seven 3' bases scored 2 points, whereas those with 4 A or T bases scored 1 point. Sequences can be classified by score as follows: 4 – class Ia, 3 – class Ib, 2, 1 or 0 – class II and -1 or -2 – class III. All knockdowns of <10% are graphed as 0.

Modifications of the Amarzgiuoui et al., Ui-Tei et al., and Takasaki et al. algorithms were applied as follows. The free energy of RNA duplex formation for 6 central bases of each shRNA (bases 6–11 of the sense strand hybridized to bases 9–14 of the antisense strand) was calculated. shRNAs with central duplex ΔGs equal to or less than -12.9 kcal/mol were assigned a minimum score (-4 for the Amarzgiuoui et al. algorithm, -2 for the Ui-Tei et al. algorithm and -13.26 for the Takasaki et al. algorithm). The scores for shRNAs with central duplex ΔGs greater than -12.9 kcal/mol were left unchanged. The cutoff value of -12.9 kcal/mol was selected empirically based upon the range of central duplex ΔGs for all shRNAs (see Table 4).

### ROC curve analysis

ROC curves were constructed as described [39]. ROC analysis requires that each shRNA is classified as either effective or ineffective. For our analyses, a shRNA was classified as effective if it reduced mRNA expression by 50% or more. A ROC curve was generated for each algorithm as follows. The decision threshold was set to one unit below the lowest shRNA score. By definition shRNAs with scores greater than or equal to the decision threshold were predicted to be effective, while those with scores less than the decision threshold were predicted to be ineffective. Then each shRNA was classified as a true positive (effective predicted to be effective), a false negative (effective predicted to be ineffective), a true negative (ineffective predicted to be ineffective) or a false positive (ineffective predicted to be effective). The true positive fraction (TPF) for the decision threshold was calculated as the number of true positives divided by the sum of the true positives and false negatives. The false positive fraction (FPF) was calculated as the number of false positives divided by the sum of the false positives and true negatives. The decision threshold was increased by one unit and the TPF and FPF calculated again. This process was repeated until the decision threshold was one unit greater than the highest scoring shRNA. ROC curves were constructed by plotting TPF versus the FPF for all decision thresholds. The area under the ROC curve was estimated by integration using the trapezoid rule.

## List of abbreviations

siRNA, small interfering RNA; shRNA, short hairpin RNA; RNAi, RNA interference; RISC, RNA-induced silencing complex; BD chemistry, BigDye Terminator v1.1 Terminator Cycle Sequencing Chemistry; dGTP chemistry, ABI Prism dGTP BigDye Terminator Cycle Sequencing Chemistry; ROC analysis, receiver operating characteristic analysis, AUC, area under the curve; TPF, true positive fraction; FPF, false positive fraction.

## Authors' contributions

DJT designed, prepared and tested shRNA vectors, conceived methods for constructing and assaying shRNA, performed analysis, interpretation, and presentation of data, and drafted the manuscript. LL performed experiments to determine optimal sequencing conditions, and assisted in the interpretation and presentation of sequencing data. JZ, BJC, HI, KLW, and JL each assisted in the construction of shRNA vectors and acquisition of data on knockdown efficiencies. JT and WR assisted in the conception, design, development and coordination of the study, and added to the intellectual content of the manuscript. WR also performed statistical analyses of shRNA efficacy.

## Supplementary Material

Additional File 1 **Effect of mixed BD:dGTP chemistries on peak resolution**. Sequencing from the 500 base region of pHSPG-shTLR2-2271, containing a hairpin structure which sequenced without problem in straight BD chemistry, is shown. Sequencing chemistries used were BD chemistry (A), 20:1 BD:dGTP chemistries (B), 10:1 BD:dGTP chemistries (C), 5:1 BD:dGTP chemistries (D) and 3:1 BD:dGTP chemistries (E). Peak resolution decreased as the amount of dGTP used increased (see boxed AAAA region at postion 475). Click here for file

Additional File 2 **Gene knockdown is similar for cell lines derived from different rounds of transduction and sorting**. Realtime data is shown for cell lines derived independently using the same viral vectors. Virus was prepared and used to transduce THP1 cells independently for each round. Values are presented as average + SEM for at least three assays run in duplicate, with the exception of shclr19.3-1504, second transduction (single value). Click here for file

## Figures and Tables

**Table 1:** ShRNA vectors prepared for this study

*accession*	*gene*	*nomenclature*	*target sequence*
[GenBank:NM_013258]	*ASC/PYCARD*	*shASC-721*	*GCTCTTCAGTTTCACACCA*
[GenBank:NM_013258]	*ASC/PYCARD*	*shASC-743*	*CCTGGAACTGGACCTGCAA*
[GenBank:AY601811]	*CLR16.2*	*shCLR16.2-482*	*GGTGAAAGCCCTCATGGAT*
[GenBank:AY601811]	*CLR16.2*	*shCLR16.2-716*	*GGGAACACGACTTCACACA*
[GenBank:AY601811]	*CLR16.2*	*shCLR16.2-3394*	*CAAATGCTCTGAAGGTAAA*
[GenBank:AY601811]	*CLR16.2*	*shCLR16.2-1630*	*GGCTGCTCAAGAAGAAATA*
[GenBank:AY116204]	*CLR19.3/NALP12*	*shCLR19.3-667*	*GTCCATGCTGGCACACAAG*
[GenBank:AY116204]	*CLR19.3/NALP12*	*shCLR19.3-991*	*GCTGCTCCCTGAGCTATCT*
[GenBank:AY116204]	*CLR19.3/NALP12*	*shCLR19.3-1504*	*GGACATCAACTGTGAGAGG*
[GenBank:AY154466]	*CLR19.6/NALP11*	*shCLR19.6-888*	*GACCTTGCAGCTGTCGAAT*
[GenBank:AY154466]	*CLR19.6/NALP11*	*shCLR19.6-1549*	*ATGGTAGACAGCTTCAAGT*
[GenBank:AY154466]	*CLR19.6/NALP11*	*shCLR19.6-2249*	*CTGACCTTATCCAGCAATC*
[GenBank:BC032474]	*MAL/TIRAP*	*shMAL-1374*	*AGGAAGTGGTACTGATCAA*
[GenBank:BC032474]	*MAL/TIRAP*	*shMAL-1504*	*TGACTCACCTGACTGATCA*
[GenBank:NM_002468]	*MYD88*	*shMYD88-1830*	*CTTTGTACCTTGATTGCCT*
[GenBank:NM_002468]	*MYD88*	*shMYD88-2207*	*ACTCACACAACAATGAACT*
[GenBank:U88878]	*TLR2*	*shTLR2-1625*	*CCATGTTACTAGTATTGAA*
[GenBank:U88878]	*TLR2*	*shTLR2-2271*	*GTATGAACTGGACTTCTCC*
[GenBank:U88880]	*TLR4*	*shTLR4-2377*	*AGGTGATTGTTGTGGTGTC*
[GenBank:U88880]	*TLR4*	*shTLR4-1923*	*CACCAGAGTTTCCTGCAAT*
[GenBank:U88880]	*TLR4*	*shTLR4-806*	*TCTGACCAATCTAGAGCAC*
[GenBank:U78798]	*TRAF6*	*shTRAF6-936*	*CCAATTCCATGCACATTCA*
[GenBank:U78798]	*TRAF6*	*shTRAF6-1326*	*GAGGAGAAACCTGTTGTGA*
[GenBank:U78798]	*TRAF6*	*shTRAF6-1563*	*GAGATAATGGATGCCAAAC*
[GenBank:AY232653]	*TRAM/TICAM2*	*shTRAM-290*	*AGAATCTGCTACAAGATGA*
[GenBank:AY232653]	*TRAM/TICAM2*	*shTRAM-482*	*TTAACAGGCAGCATAAATA*
[GenBank:AB086380]	*TRIF/TICAM1*	*shTRIF-1786*	*AGAGCTACTTGTCCTACCA*

**Table 2:** Evaluation of sequencing results of three DNA hairpin constructs. Average ratio of peak height after to before the hairpin region was determined as a measure of how well the sequence read through the hairpin structure. The greater the peak height ratio, the greater the ability to sequence through the hairpin. A value of 1 indicates no loss in peak height, and a value of zero indicates a complete stop in sequence after the hairpin region. All values are the averages of at least triplicate sequencing reactions.

		**DNA Plasmid**		
		**pHSPG-shTLR4**	**pHSPG-shmutTLR4**	**pHSPG-shmCNN3**

**Chemistry**	**DNA relaxing agent**	**Peak Height Ratio**	**Peak Height Ratio**	**Peak Height Ratio**

BDv1.1	None	0.0	0.4	0.0
BDv1.1	5% DMSO	0.0	0.6	0.2
BDv1.1	0.83 M Betaine	0.3	0.9	0.7
BDv1.1	1 × PCRx Enhancer	0.1	0.7	0.3
BDv1.1	0.83 M Betaine & 1 × PCRx Enhancer	0.6	0.9	0.5
20:1 BD:dGTP	None	0.4	0.6	0.5
20:1 BD:dGTP	5% DMSO	0.6	0.7	0.6
20:1 BD:dGTP	0.83 M Betaine	0.7	1.0	0.8
20:1 BD:dGTP	1 × PCRx Enhancer	0.6	0.8	0.7
20:1 BD:dGTP	0.83 M Betaine & 1 × PCRx Enhancer	0.8	0.9	0.8
10:1 BD:dGTP	None	0.5	0.6	0.6
10:1 BD:dGTP	5% DMSO	0.7	0.8	0.7
10:1 BD:dGTP	0.83 M Betaine	0.8	0.9	0.9
10:1 BD:dGTP	1 × PCRx Enhancer	0.7	0.8	0.8
10:1 BD:dGTP	0.83 M Betaine & 1 × PCRx Enhancer	0.9	1.0	0.9
10:1 BD:dGTP	1 × ThermoFidelase I	0.1	0.2	0.2
5:1 BD:dGTP	None	0.6	0.7	0.7
3:1 BD:dGTP	None	0.6	0.6	0.6
dGTP only	None	0.7	0.7	0.8

**Table 3:** Comparison of knockdown efficacy and siRNA design algorithm. Average knockdown was measured by real-time PCR of triplicate samples. All averages are accurate within 10% SEM. Asterisks indicate high Takasaki et al. algorithm scores that have poor corresponding knockdown efficacy.

**1**	**2**	**3**	**4**	**5**	**6**	**7**	**8**	**9**
**nomenclature**	**cell tested**	**% knockdown**	**5' ΔΔG**	**Reynolds**	**Hsieh**	**Amarzguioui**	**Ui-Tei**	**Takasaki**

*shASC-721*	THP1	91	0.65	6	0	2	2	8.07
*shASC-743*	THP1	74	-2.86	5	2	4	2	7.33
*shCLR16.2-482*	jurkat	37	-1.42	5	3	4	3	10.1 *
*shCLR16.2-716*	jurkat	64	-1.68	6	0	4	3	5.05
*shCLR16.2-3394*	jurkat	74	1.06	9	2	3	4	0
*shCLR16.2-1630*	jurkat	59	-8.1	8	1	5	4	4.81
*shCLR19.3-667*	THP1	71	-1.59	3	1	1	1	10.1
*shCLR19.3-991*	THP1	13	-4.62	5	2	3	3	10.1 *
*shCLR19.3-1504*	THP1	70	1.06	4	0	0	0	8.42
*shCLR19.6-888*	jurkat	<10%	-0.33	5	2	2	2	3.78
*shCLR19.6-1549*	jurkat	<10%	-2.13	5	1	2	1	-2.6
*shCLR19.6-2249*	jurkat	<10%	-2.22	4	3	4	3	9.8 *
*shMAL-1374*	THP1	69	-1.86	8	0	2	2	-11
*shMAL-1504*	THP1	65	-1.54	8	0	0	1	-3.8
*shMYD88-1830*	THP1	73	3.06	5	3	1	3	2.7
*shMYD88-2207*	THP1	53	-1.31	5	2	1	2	-4
*shTLR2-1625*	THP1	59	-3.85	9	0	3	4	3.13
*shTLR2-2271*	THP1	57	1.84	4	1	1	0	14.7
*shTLR4-2377*	THP1	79	0.61	4	0	1	-2	-6.6
*shTLR4-1923*	THP1	<10%	-2.58	6	1	4	3	-0.1
*shTLR4-806*	THP1	<10%	1.52	4	2	-2	-2	-0.7
*shTRAF6-936*	THP1	51	-0.18	9	2	3	4	0
*shTRAF6-1326*	THP1	62	-2.83	5	2	3	3	13.1
*shTRAF6-1563*	THP1	53	0.26	3	0	3	0	7.38
*shTRAM-290*	THP1	<10%	1.18	8	0	1	2	-8.9
*shTRAM-482*	THP1	<10%	-1.92	9	2	1	2	-1.4
*shTRIF-1786*	THP1	<10%	-0.99	8	1	0	0	1.03

**Table 4:** Modification of algorithm scores based upon shRNA central duplex ΔG. The percent knockdown data represents the average knockdown as shown in Table 3. shRNAs with a central ΔG equal to or less than -12.9 kcal/mol are underlined. These were assigned minimum scores according to the algorithm modification. Minimum scores are: Amarzguioui et al. algorithm, -4; Ui-Tei et al. algorithm, -2; Takasaki et al. algorithm, -13.26. The three shRNAs that scored high in the original Takasaki et al. algorithm but have poor knockdown efficacy are marked with asterisks. The modification minimized scoring for these shRNAs, thus increasing specificity of the algorithm.

**Nomenclature**	**% knockdown**	**Central ΔG**	**Modified**	**Modified**	**Modified**
			
			**Amarzguioui**	**Ui-Tei**	**Takasaki**
*shASC-721*	91	-9.7	2	2	8.07
*shASC-743*	74	-10.6	4	2	7.33
*shCLR16.2-482*	37	-12.9	-4	-2	-13.26 *
*shCLR16.2-716*	64	-11.3	4	3	5.05
*shCLR16.2-3394*	74	-12	3	4	0
*shCLR16.2-1630*	59	-9.6	5	4	4.81
*shCLR19.3-667*	71	-13	-4	-2	-13.26
*shCLR19.3-991*	13	-13.1	-4	-2	-13.26 *
*shCLR19.3-1504*	70	-10.9	0	0	8.42
*shCLR19.6-888*	<10%	-7.8	2	1	-2.6
*shCLR19.6-1549*	<10%	-13.1	-4	-2	-13.26 *
*shCLR19.6-2249*	<10%	-10.9	2	2	3.78
*shMAL-1374*	69	-11.2	2	2	-11
*shMAL-1504*	65	-11.8	0	1	-3.8
*shMYD88-1830*	73	-9.8	1	3	2.7
*shMYD88-2207*	53	-9.6	1	2	-4
*shTLR2-1625*	59	-7.1	3	4	3.13
*shTLR2-2271*	57	-10.6	1	0	14.7
*shTLR4-2377*	79	-7.3	1	-2	-6.6
*shTLR4-1923*	<10%	-8.5	4	3	-0.1
*shTLR4-806*	<10%	-9.8	-2	-2	-0.7
*shTRAF6-936*	51	-10.9	3	4	0
*shTRAF6-1326*	62	-9.7	3	3	13.1
*shTRAF6-1563*	53	-9.8	3	0	7.38
*shTRAM-290*	<10%	-11	1	2	-8.9
*shTRAM-482*	<10%	-12.9	-4	-2	-13.26
*shTRIF-1786*	<10%	-8.69	0	0	1.03

**Table 5:** Previously published shRNA sequences analyzed in this study

**accession**	**gene**	**target sequence**	**% knockdown**	**reference**
[GenBank:NM_002737]	PKCalpha	GAACAACAAGGAATGACTT	90	[27]
[GenBank:NM_002738]	PKCbeta1	GGAAGCTGTGGCCATCTGC	90	[27]
[GenBank:NM_006257]	PKCtheta	TTGGATGAGGTGGATAAAA.	90	[27]
[GenBank:AY599018]	HBV1	GGAGGTTGGGGACTGCGAA	75	[28]
[GenBank:DQ207798]	HBV2	CAAGGCACAGCTTGGAGGC	61	[28]
[GenBank:DQ207798]	HBV3	CGAGGCGAGGGAGTTCTTC	63	[28]
[GenBank:NM_012029]	Ecsit	GCTGTGGTTCACCCGATTC	0	[29]
[GenBank:NM_012029]	Ecsit	GGTCACTGTCTACCAGATG	95	[29]
[GenBank:U47298]	Firefly Luciferase	GTTGGCAGAAGCTATGAAA	93	[18]
[GenBank:U47298]	Firefly Luciferase	GATTTCGAGTCGTCTTAAT	96	[18]
[GenBank:U47298]	Firefly Luciferase	GCACTCTGATTGACAAATA	93	[18]
[GenBank:U47298]	Firefly Luciferase	GACAAATACGATTTATCTA	86	[18]
[GenBank:U47298]	Firefly Luciferase	GATTATGTCCGGTTATGTA	97	[18]
[GenBank:U47298]	Firefly Luciferase	GGATGGATGGCTACATTCT	92	[18]
[GenBank:U47298]	Firefly Luciferase	GCCTGAAGTCTCTGATTAA	95	[18]
[GenBank:U47298]	Firefly Luciferase	AACATAAAGAAAGGCCCGG	0	[18]
[GenBank:U47298]	Firefly Luciferase	GTCGCTCTGCCTCATAGAA	72	[18]
[GenBank:U47298]	Firefly Luciferase	GATTTCGAGTCGTCTTAAT	98	[18]
[GenBank:U47298]	Firefly Luciferase	AATCTTGTAATCCTGAAGG	94	[18]
[GenBank:NM_001025257]	VEGF	GCTACTGCCGTCCAATTGA	26.4	[30]
[GenBank:AY500353]	VEGF	GGCGAGGCAGCTTGAGTTA	14.7	[30]
[GenBank:NM_001025250]	VEGF	AATCAGTTCGAGGAAAGGG	42.7	[30]
[GenBank:NM_000546]	p53	GTCTGTGACTTGCACGTAC	95	[31]
[GenBank:NM_000546]	p53	GCAGTCACAGCACATGACG	85	[31]
[GenBank:NM_000546]	p53	GACTCCAGTGGTAATCTAC	87	[31]
[GenBank:NM_009820]	Runx2i	CGGGCTCACGTCGCTCATC	55	[32]
[GenBank:AM075811]	HCV-38	CACTCCCCTGTGAGGAACT	28	[33]
[GenBank:AM075811]	HCV-56	TACTGTCTTCACGCAGAAA	38	[33]
[GenBank:AM075811]	HCV-71	GAAAGCGTCTAGCCATGGC	0	[33]
[GenBank:AM075811]	HCV-138	CCATAGTGGTCTGCGGAAC	92	[33]
[GenBank:AM075811]	HCV-156	CCGGTGAGTACACCGGAAT	28	[33]
[GenBank:AM075811]	HCV-174	TTGCCAGGACGACCGGGTC	0	[33]
[GenBank:AM075811]	HCV-279	CCTTGTGGTACTGCCTGAT	76	[33]
[GenBank:AM075811]	HCV-301	GTGCTTGCGAGTGCCCCGG	49	[33]
[GenBank:AM075811]	HCV-321	AGGTCTCGTAGACCGTGCA	94	[33]
[GenBank:AM075811]	HCV-334	CGTGCACCATGAGCACGAA	91	[33]
[GenBank:AM075811]	HCV-360	CCTCAAAGAAAAACCAAAC	91	[33]
[GenBank:AM075811]	HCV-5879	GGTGCTTGTGGATATTTTG	68	[33]

## References

[B1] Matzke MA, Birchler JA (2005). RNAi-mediated pathways in the nucleus. Nat Rev Genet.

[B2] Huppi K, Martin SE, Caplen NJ (2005). Defining and assaying RNAi in mammalian cells. Mol Cell.

[B3] Paddison PJ, Caudy AA, Bernstein E, Hannon GJ, Conklin DS (2002). Short hairpin RNAs (shRNAs) induce sequence-specific silencing in mammalian cells. Genes Dev.

[B4] Sui G, Soohoo C, Affar el B, Gay F, Shi Y, Forrester WC (2002). A DNA vector-based RNAi technology to suppress gene expression in mammalian cells. Proc Natl Acad Sci U S A.

[B5] Brummelkamp TR, Bernards R, Agami R (2002). A system for stable expression of short interfering RNAs in mammalian cells. Science.

[B6] Paddison PJ, Caudy AA, Sachidanandam R, Hannon GJ (2004). Short hairpin activated gene silencing in mammalian cells. Methods Mol Biol.

[B7] Wong AW, Brickey WJ, Taxman DJ, van Deventer HW, Reed W, Gao JX, Zheng P, Liu Y, Li P, Blum JS, McKinnon KP, Ting JP (2003). CIITA-regulated plexin-A1 affects T-cell-dendritic cell interactions. Nat Immunol.

[B8] Tomar RS, Matta H, Chaudhary PM (2003). Use of adeno-associated viral vector for delivery of small interfering RNA. Oncogene.

[B9] Rubinson DA, Dillon CP, Kwiatkowski AV, Sievers C, Yang L, Kopinja J, Rooney DL, Ihrig MM, McManus MT, Gertler FB, Scott ML, Van Parijs L (2003). A lentivirus-based system to functionally silence genes in primary mammalian cells, stem cells and transgenic mice by RNA interference. Nat Genet.

[B10] Moore MD, McGarvey MJ, Russell RA, Cullen BR, McClure MO (2005). Stable inhibition of hepatitis B virus proteins by small interfering RNA expressed from viral vectors. J Gene Med.

[B11] Tran N, Cairns MJ, Dawes IW, Arndt GM (2003). Expressing functional siRNAs in mammalian cells using convergent transcription. BMC Biotechnol.

[B12] Wadhwa R, Kaul SC, Miyagishi M, Taira K (2004). Vectors for RNA interference. Curr Opin Mol Ther.

[B13] Khvorova A, Reynolds A, Jayasena SD (2003). Functional siRNAs and miRNAs exhibit strand bias. Cell.

[B14] Schwarz DS, Hutvagner G, Du T, Xu Z, Aronin N, Zamore PD (2003). Asymmetry in the assembly of the RNAi enzyme complex. Cell.

[B15] Reynolds A, Leake D, Boese Q, Scaringe S, Marshall WS, Khvorova A (2004). Rational siRNA design for RNA interference. Nat Biotechnol.

[B16] Saetrom P, Snove OJ (2004). A comparison of siRNA efficacy predictors. Biochem Biophys Res Commun.

[B17] Takasaki S, Kotani S, Konagaya A (2004). An effective method for selecting siRNA target sequences in mammalian cells. Cell Cycle.

[B18] Ui-Tei K, Naito Y, Takahashi F, Haraguchi T, Ohki-Hamazaki H, Juni A, Ueda R, Saigo K (2004). Guidelines for the selection of highly effective siRNA sequences for mammalian and chick RNA interference. Nucleic Acids Res.

[B19] Hsieh AC, Bo R, Manola J, Vazquez F, Bare O, Khvorova A, Scaringe S, Sellers WR (2004). A library of siRNA duplexes targeting the phosphoinositide 3-kinase pathway: determinants of gene silencing for use in cell-based screens. Nucleic Acids Res.

[B20] Amarzguioui M, Prydz H (2004). An algorithm for selection of functional siRNA sequences. Biochem Biophys Res Commun.

[B21] Jackson AL, Linsley PS (2004). Noise amidst the silence: off-target effects of siRNAs?. Trends Genet.

[B22] Paddison PJ, Cleary M, Silva JM, Chang K, Sheth N, Sachidanandam R, Hannon GJ (2004). Cloning of short hairpin RNAs for gene knockdown in mammalian cells. Nat Methods.

[B23] Miller VM, Xia H, Marrs GL, Gouvion CM, Lee G, Davidson BL, Paulson HL (2003). Allele-specific silencing of dominant disease genes. Proc Natl Acad Sci U S A.

[B24] Ducat DC, Herrera FJ, Triezenberg SJ (2003). Overcoming obstacles in DNA sequencing of expression plasmids for short interfering RNAs. Biotechniques.

[B25] Malykh A, Malykh O, Polushin N, Kozyavkin S, Slesarev A (2004). Finishing "working draft" BAC projects by directed sequencing with ThermoFidelase and Fimers. Methods Mol Biol.

[B26] Slesarev AI, Mezhevaya KV, Makarova KS, Polushin NN, Shcherbinina OV, Shakhova VV, Belova GI, Aravind L, Natale DA, Rogozin IB, Tatusov RL, Wolf YI, Stetter KO, Malykh AG, Koonin EV, Kozyavkin SA (2002). The complete genome of hyperthermophile Methanopyrus kandleri AV19 and monophyly of archaeal methanogens. Proc Natl Acad Sci U S A.

[B27] Trushin SA, Pennington KN, Carmona EM, Asin S, Savoy DN, Billadeau DD, Paya CV (2003). Protein kinase Calpha (PKCalpha) acts upstream of PKCtheta to activate IkappaB kinase and NF-kappaB in T lymphocytes. Mol Cell Biol.

[B28] Zhang XN, Xiong W, Wang JD, Hu YW, Xiang L, Yuan ZH (2004). siRNA-mediated inhibition of HBV replication and expression. World J Gastroenterol.

[B29] Xiao C, Shim JH, Kluppel M, Zhang SS, Dong C, Flavell RA, Fu XY, Wrana JL, Hogan BL, Ghosh S (2003). Ecsit is required for Bmp signaling and mesoderm formation during mouse embryogenesis. Genes Dev.

[B30] Zhang L, Yang N, Mohamed-Hadley A, Rubin SC, Coukos G (2003). Vector-based RNAi, a novel tool for isoform-specific knock-down of VEGF and anti-angiogenesis gene therapy of cancer. Biochem Biophys Res Commun.

[B31] Liu XD, Ma SM, Liu Y, Liu SZ, Sehon A (2004). Short hairpin RNA and retroviral vector-mediated silencing of p53 in mammalian cells. Biochem Biophys Res Commun.

[B32] Inman CK, Shore P (2003). The osteoblast transcription factor Runx2 is expressed in mammary epithelial cells and mediates osteopontin expression. J Biol Chem.

[B33] Kronke J, Kittler R, Buchholz F, Windisch MP, Pietschmann T, Bartenschlager R, Frese M (2004). Alternative approaches for efficient inhibition of hepatitis C virus RNA replication by small interfering RNAs. J Virol.

[B34] Chalk AM, Wahlestedt C, Sonnhammer EL (2004). Improved and automated prediction of effective siRNA. Biochem Biophys Res Commun.

[B35] Yiu SM, Wong PW, Lam TW, Mui YC, Kung HF, Lin M, Cheung YT (2005). Filtering of ineffective siRNAs and improved siRNA design tool. Bioinformatics.

[B36] Huesken D, Lange J, Mickanin C, Weiler J, Asselbergs F, Warner J, Meloon B, Engel S, Rosenberg A, Cohen D, Labow M, Reinhardt M, Natt F, Hall J (2005). Design of a genome-wide siRNA library using an artificial neural network. Nat Biotechnol.

[B37] Meissner EG, Coffield VM, Su L (2005). Thymic pathogenicity of an HIV-1 envelope is associated with increased CXCR4 binding efficiency and V5-gp41-dependent activity, but not V1/V2-associated CD4 binding efficiency and viral entry. Virology.

[B38] Xia T, SantaLucia JJ, Burkard ME, Kierzek R, Schroeder SJ, Jiao X, Cox C, Turner DH (1998). Thermodynamic parameters for an expanded nearest-neighbor model for formation of RNA duplexes with Watson-Crick base pairs. Biochemistry.

[B39] Riffenburgh RH (1999). Statistics in Medicine.

